# Smile Reproducibility and Its Relationship to Self-Perceived Smile Attractiveness

**DOI:** 10.3390/biology11050719

**Published:** 2022-05-07

**Authors:** Denitsa Dobreva, Nikolaos Gkantidis, Demetrios Halazonetis, Carlalberta Verna, Georgios Kanavakis

**Affiliations:** 1 Department of Pediatric Oral Health and Orthodontics, University Center for Dental Medicine UZB, University of Basel, Mattenstrasse 40, 4058 Basel, Switzerland; denitsa.dobreva@unibas.ch (D.D.); carlalberta.verna@unibas.ch (C.V.); 2 Department of Orthodontics and Dentofacial Orthopedics, University of Bern, 3001 Bern, Switzerland; nikolaos.gkantidis@zmk.unibe.ch; 3 Department of Orthodontics, School of Dentistry, National and Kapodistrian University of Athens, GR-11527 Athens, Greece; dhalaz@dent.uoa.gr; 4 Department of Orthodontics, Tufts University School of Dental Medicine, Boston, MA 02111, USA

**Keywords:** smile reproducibility, lip morphology, 3dMD, facial expressions, 3D imaging, stereophotogrammetry, smile attractiveness

## Abstract

**Simple Summary:**

The smile plays an important role in personal, professional, and romantic relationships among humans. A smile may be posed or spontaneous, based on the external stimulus, and is characterised by a combination of facial movements that form the “smiling face”. Although the reproducibility of facial expressions, including the smile, has been studied before there are no reports on the reproducibility of the lip morphology upon smiling. Here, we assess a group of young adults who volunteered to pose for a social smile at two time points, four weeks apart. At the same visit they were also asked to assess the attractiveness of their own smile. Our results show that lip morphology during smiling is highly consistent among young adults. Females presented higher consistency in the shape of the smile, including lip morphology, compared to males. Self-perceived smile attractiveness was not associated to smile consistency.

**Abstract:**

The reproducibility of facial expressions has been previously explored, however, there is no detailed information regarding the reproducibility of lip morphology forming a social smile. In this study, we recruited 93 young adults, aged 21–35 years old, who agreed to participate in two consecutive study visits four weeks apart. On each visit, they were asked to perform a social smile, which was captured on a 3D facial image acquired using the 3dMD camera system. Assessments of self-perceived smile attractiveness were also performed using a VAS scale. Lip morphology, including smile shape, was described using 62 landmarks and semi-landmarks. A Procrustes superimposition of each set of smiling configurations (first and second visit) was performed and the Euclidean distance between each landmark set was calculated. A linear regression model was used to test the association between smile consistency and self-perceived smile attractiveness. The results show that the average landmark distance between sessions did not exceed 1.5 mm, indicating high repeatability, and that females presented approximately 15% higher smile consistecy than males (*p* < 0.05). There was no statistically significant association between smile consistency and self-perceived smile attractiveness (η^2^ = 0.015; *p* = 0.252), when controlling for the effect of sex and age.

## 1. Introduction

Facial expressions lay the foundation of interpersonal communication [1]. They are not only specific to humans but are also observed in animals [2]. There is a large spectrum of facial expressions most of which reflect emotions, mood, sensations, and either complement or substitute verbal expressions. Some facial expressions are undetermined and cannot be easily interpreted [3], while the lack of facial expression either signifies pathology (for example in the Moebius syndrome [4]) or creates uncertainty during human interaction [5]. The main facial expressions are happiness, anger, disgust, fear, contempt, and surprise [6,7].

Smiling is the most common facial expression. It is linked to a variety of pleasant emotions and creates positive perceptions upon interaction with other humans [8]. Smiling is characterised by specific morphological changes of the facial structures [9]. The most prominent feature is the change in the peri-oral region, with the lip corners pulling up and backwards in order to expose the teeth [10,11]. This also leads to elongation of the chin region and deepening of the nasolabial grooves [12]. The lower third of the face is not affected in isolation to the rest of the face upon smiling. Significant changes also appear around the periorbital region [7,13,14].

A smile is usually perceived as an expression of happiness or joy. However, depending on the social situation, a smile is also used to create a certain perception [15]. For instance, in greetings or conversations it is a sign of politeness [8], a tool to ascertain positive social connections [16] or mask a feeling of embarrassment. On the other hand, a smile can establish dominance or even hide ill intent [17,18].

There are two main types of smiles: genuine or spontaneous smiles and social or evoked ones [1,19]. Spontaneous smiles are a response to a pleasant stimulus and are not controlled. A social smile, on the other hand, is a posed smile, one that is consciously performed in a social environment during interpersonal communication or upon request [14]. These two types are triggered by different facial muscle activity, as shown by research using electrodes to measure muscle activity during various facial expressions [20,21]. In social sciences, the differences between a spontaneous and an evoked smile have been studied in an attempt to identify deception in a human face [22,23,24]. A third type of smile, namely the “negative affect smile” has also been described [7]. This is often observed in patients with unipolar depression and is characterized by activation of muscle groups specific to negative emotions [13].

From an evolutionary standpoint, humans have learned to use their smile in their favour, in order to adapt to various social instances [16], suppress, neutralize or amplify their emotions, making it difficult to be read by other individuals [25]. Being able to understand emotions, on the other hand, poses an advantage in human communication and improves the perceived impression of other individuals [17,25]. The inability to smile or to perceive the smile of others are sings of psychological pathology and detrimental for building human relationships [26,27].

Despite the large body of evidence underpinning the impact that a smile has in social and interpersonal relationships, there is scattered information regarding the reproducibility of a social smile. Previous investigations have explored the reproducibility of facial expressions, including the smile. However they have only assessed changes in the entire face and have mostly studied small sample sizes [28,29,30]. There are no reports focusing on the repeatability of the perioral tissue morphology upon a social smile and therefore the primary aim of this study was to assess the reproducibility of a social smile and the associated lip morphology using three-dimensional surface data of the perioral structures.

Furthermore, smiling is among the main determinants of facial attractiveness and creates perception of happiness and youth during human interaction [31,32]. Therefore, it can be assumed that individuals who consider themselves as having an attractive smile might tend to smile more consistently, in order to always create similar positive impressions within a social environment. However, it is not known if there is an association between the consistency of a social smile and its self-perceived attractiveness, and thus this assumption was also evaluated here.

## 2. Materials and Methods

### 2.1. Ethical Approval

This study was reviewed and approved by the Health Sciences Institutional Review Board (IRB) of Tufts University in Boston, MA, USA (IRB#: 11181).

#### Study Sample

The study population consisted of 93 (36 males; 57 females) young adults, who were all pre-doctoral students at Tufts University in Boston, MA, USA. All participants were aged 21 to 35 years, were raised in the USA, and had various ethnic backgrounds. Their participation was compensated with a $20 gift card. This group is part of a larger study population, recruited for investigating the association between facial morphology and self-perceived attractiveness [33,34].

### 2.2. 3D Facial Image Acquisition

The volunteers were asked to participate in two consecutive image acquisition sessions four weeks apart. Three-dimensional facial images were captured using a stereophotogrammetry system (3dMD, Atlanta, GA, USA), which has a spatial resolution of less than 0.5 mm and a geometry accuracy of less than 0.2 mm [35,36,37]. Subjects were positioned at a fixed distance of approximately 100 cm from the camera unit, with their head slightly raised (10 degrees) according to the camera guidelines (upright head position). To ensure standardization, participants were seated on a chair placed at the correct distance, with their upper body in a comfortable upright position and their back resting on the back of the chair. The participants were asked to perform a social smile expression, beginning with the rest position, and ending with the rest position again to ensure that all phases of the facial movement were recorded—rest, onset, apex, offset, rest. At the end of both study visits, 186 surface images depicting the social smile were collected and were used in the present study.

### 2.3. Smile Shape Definition

**Figure 1:**
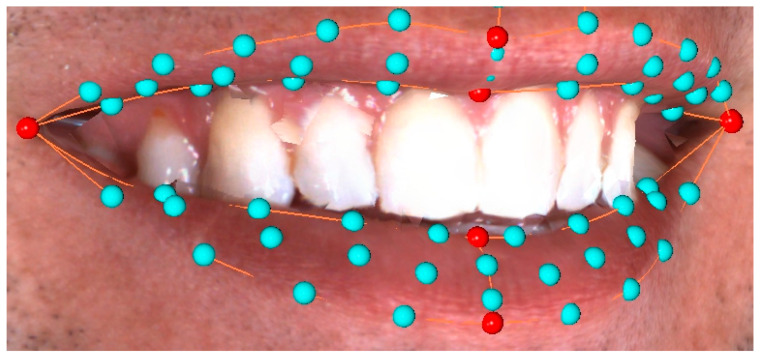
Distribution of fixed (red) and sliding (light blue) landmarks along the lip curvatures and the upper and lower lip surfaces. Note that the inner stomia coincide with the outer stomia in this image.

All three-dimensional images were imported into “ViewBox 4.1” software (dHAL Software, Kifissia, Greece) for further processing. This was done in two phases; first, the images were cropped to reduce artefacts and then digitized, using a pre-determined template. Digitization was performed by the same operator, according to a previously described protocol [33]. Smile shape was described with 62 landmarks. The centre of the upper and lower stomia, the right and left outer and inner stomia and the upper and lower midpoint of the outer lip curvature were regarded as fixed landmarks, while all other landmarks were handled as semi-landmarks and were allowed to slide along their respective curves or surfaces. In the first step of the digitization process, the outer and inner outlines of the mouth were delineated with curves that were placed manually along the anatomical boundaries of the upper and lower lips. In the second step, all 62 landmarks were automatically placed on these curves. The fixed landmarks were the ones placed at the extremes or at the midpoints of the curvatures, and the semi-landmarks were placed at equidistant positions along the upper and lower lip curvatures or on the surface of the upper and lower lip (Figure 1).

After completion of the digitization process, the variation in landmark configurations was reduced through an iterative sliding process. First, an average landmark configuration was created from all three-dimensional surface images. Since semi-landmarks do not have a biological interpretation, and are thus not homologous among various subjects, they were then allowed to slide along their respective curves or surfaces in order to create more homologous landmarks as related to the average shape configuration. During the first round of sliding, landmarks were allowed to slide and were reprojected six times in order to reduce the amount of bending energy between the individual landmark configurations and the average configuration. After the initial sliding, a new average configuration was created and the entire sliding process was repeated to further reduce bending energy between the new individual configurations and the new average landmark configuration. This iterative process was repeated three times in total until bending energy was minimized and the best possible homology between landmarks was achieved [38,39]. The resulting landmark configurations were considered to be homologous representations of the lip surfaces and comprised the final sample for all following shape analyses.

Following the sliding process, a pairwise partial Procrustes Superimposition was performed between corresponding sets of landmark configurations, in order to minimize the sum of squared distances between all individual landmarks [40]. Each set comprised the landmark configurations of the first and second study visits for each subject. The resulting shape coordinates were then extracted into an Excel worksheet (Microsoft Excel, Microsoft ©, Redmond, WA, USA) for further analysis. For each subject (i.e., for each set of smile configurations), the Euclidean distance between each of the 62 landmarks was calculated. In addition, the average distance of all landmarks for each subject and the average distance at each landmark were also generated.

### 2.4. Self-Perceived Smile Attractiveness

In addition to image acquisition, each individual filled out a questionnaire with basic demographic information and then performed a self-assessment of their self-perceived smile attractiveness on a 100 mm visual analogue scale (VAS) [41]. The question asked was: “How would you rate the aesthetic appearance of your smile?”, with possible scores ranging between “completely unattractive” (left extreme on the scale) and “extremely attractive” (right extreme on the scale). Participants were instructed to place a vertical mark on the VAS scale to indicate their answer. The distance (in mm) between the left extreme end of the scale and their mark indicated participants’ answers to the study question. The exact process of collecting information from the questionnaire has been previously described in detail [33,34].

### 2.5. Statistical Analyses

The average change at each of the 62 landmarks describing the smile as well as the direction of this change were presented visually with the use of graphics created with Viewbox 4 software, version 4.1.0.1 Beta 64 (dHal Software, Kifissia, Greece) and Powerpoint (Microsoft Powerpoint, Microsoft ©, Redmond, WA, USA). The association between change in smile shape, namely smile consistency, and self-perceived smile attractiveness was assessed with a univariate regression model, with self-perceived smile attractiveness being the dependent variable and sex, age and smile consistency being the predictor variables. The regression model was run in SPSS version 27.0 (IBM, Armonk, NY, USA). Differences in smile consistency and self-perceived smile attractiveness between males and females were evaluated with Student’s t-test for independent samples and data were presented with descriptive statistics. For all analyses, a type-I error of 5% was accepted.

## 3. Results

### 3.1. Error Calculation

In order to assess systematic and random error of the digitization process, a Procrustes superimposition was performed between the smile shapes from two repeated digitisations of the same smiles (*n* = 20). Systematic error was assessed through permutation tests (100,000 permutations) on the mean Procrustes distance between first and second digitizations and was found to be non-significant, indicating no systematic error (*p* = 0.999). Random digitization error was determined as the percentage of total variance in shape space, according to a previously reported method [42]. This resulted in a random error of 6.8%, which represented the amount of variance attributed to the repetition of the entire digitization process and was considered to be acceptable. Error assessment confirmed good intra-rater reliability throughout the study. In addition, the error related to the questionnaire answers was evaluated through Bland-Altman tests and was found to be negligible. The detailed results of the Bland Altman tests are presented elsewhere [33].

### 3.2. Smile Consistency

**Figure 2:**
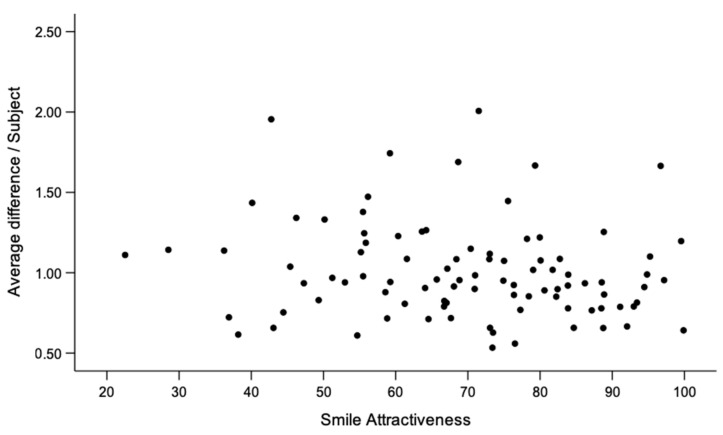
Scatterplot showing the association between smile consistency and self-perceived smile attractiveness. The y-axis represents the average landmark difference per individual between first and second smile and the x-axis represents the average score of each individual in self-perceived smile attractiveness.

The variability in landmark position in shape space revealed that smile shape was consistent, on average, between the first and second visits. The scatterplot in Figure 2 shows that in most subjects the average Euclidean distance between all landmarks from the first to the second visit did not exceed 1.5 mm. Females presented a higher smile consistency than males and showed higher scores in self-perceived smile attractiveness (Table 1).

**Figure 3:**
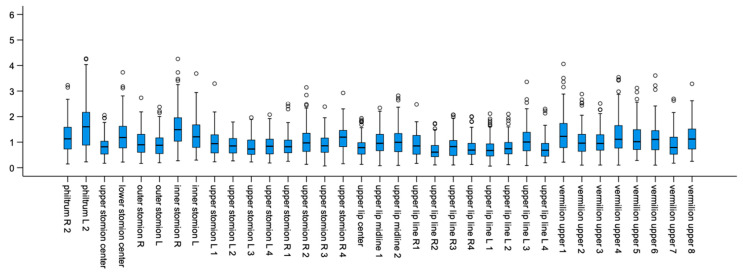
Box plots showing the average and the range of differences (in mm) per upper lip point in milimeters.

**Figure 4:**
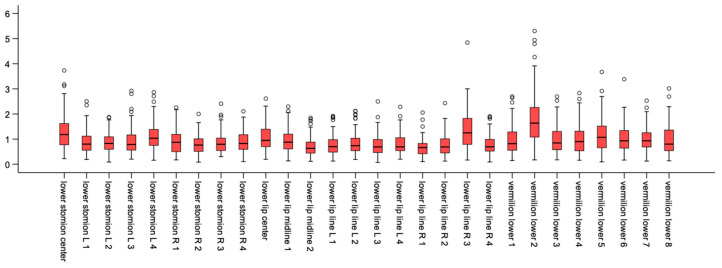
Box plots showing the average and the range of differences (in mm) per lower lip point in millimeters.

**Figure 5:**
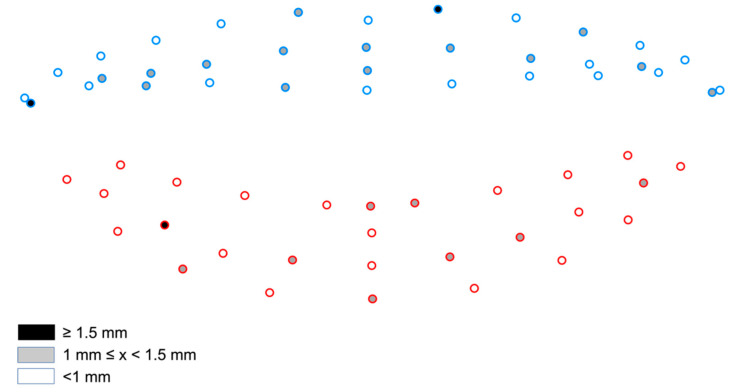
Color-coded map of the magnitude of differences between repeated social smiles at each landmark. (Blue: Upper lip landmarks / Red: Lower lip landmarks).

**Figure 6:**
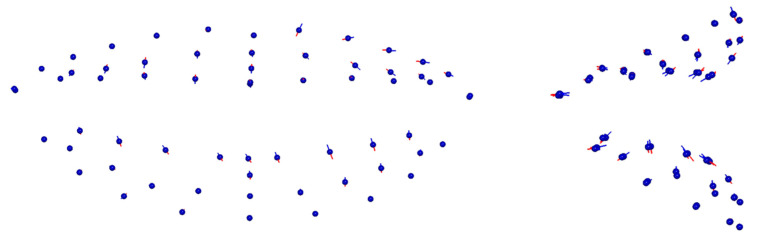
Variability and direction of change at each smile landmark.

The median difference, as well as the range of recorded differences, at each landmark is presented with Box-plots in Figure 3 and Figure 4, for the upper lip points and the lower lip points, respectively. The empty circles beyond the maximum values correspond to outliers in the sample. Average differences between corresponding landmarks ranged from 0.70 mm to 1.98 mm, with larger differences being observed in the upper lip. A more clinically interpretable depiction of the magnitude of differences at each landmark can be seen in Figure 5. The areas of the lips that showed larger differences (i.e., smaller consistency) were the upper and lower lip vermillia. The variability and direction of differences at each smile landmark is presented in Figure 6. The areas of largest variability were the upper and lower stomia.

### 3.3. Smile Consistency and Self-Perceived Smile Attractiveness

The applied regression model did not show a statistically significant association between smile consistency and self-perceived smile attractiveness in the present sample (η^2^ = 0.015; *p* = 0.252), when controlling for the effect of sex and age. In other words, individuals with less variation in the position of smile landmarks between repeated social smiles did not present larger VAS scores than individuals with larger differences between corresponding landmarks.

## 4. Discussion

This study assessed the reproducibility of a social smile in a sample of young adults using three-dimensional imaging and geometric morphometric methods to describe smile shape, including lip morphology. The results showed that social smiles are highly reproducible in all three dimensions in females and males, with females showing higher consistency.

The reproducibility of facial expressions has been studied previously. However, here we focus solely on lip morphology and associated changes in smile shape. Until now, studies have evaluated the reproducibility of the entire smiling facial expression, briefly capturing the perioral tissue morphology [28,29,30]. This provides valuable information since human interaction is affected by overall facial expressions instead of isolated facial parts. Nevertheless, social smiling does not only occur spontaneously, but is also a learned trait, and thus, the inclusion of other facial features in the assessment may skew the outcomes concerning the social smile as a distinct feature. Therefore, it may be argued that studying smile reproducibility by examining the entire face does not necessarily correspond to the reproducibility seen purely in the smile.

Furthermore, the present study performed a thorough assessment of the reproducibility of the social smile. According to the facial action coding system (FACS) smiling is related to action units (AU) 6 and 12, which are associated with a contraction of the orbicularis and zygomaticus muscles, respectively. A posed smile usually involves AU12, which is easily controlled, extends the width of the mouth laterally and lifts its corners to create a U-shape. A genuine smile involves both AU12 and AU6 and thus also involves a lifting of the cheeks [43]. In addition, a posed smile is more asymmetric than a genuine smile and presents larger variability in the displacement of the mouth corners [8,14]. However, in 20–70% of cases posed smiles simulate the expression seen in genuine smiles [44,45,46,47]. With the present methodology, any variation in facial parts other than the lips, which might derive from the factors described above and affect the outcomes was excluded. The results of the present study support previous findings indicating that regardless of the apparent individuality in performing a posed smile, the lip morphology defining the social smile shape remains consistent. Although the landmarks used to describe lip morphology correspond to different areas of the lips and showed different amounts of variability in their movement, the interpretation of the results did not focus on the individual movement at each landmark, since there was no biological justification for such an interpretation. The movement of the lips upon social smiling is mainly controlled by the zygomaticus muscle, as described above, and thus they are moved together. Electromyography studies that have evaluated muscle fatigue after multiple repetitions of voluntary facial expressions have found that the muscles associated with the smiling expression show a 24% decrease in muscle activity after repetitive activation, which is notably lower than muscles controlling other facial movements such as brow raise (42% decrease) and lip pucker (29% decrease) [48]. Maintaining the magnitude of muscular activity on repetitive smiling expressions might be a factor contributing to the smile consistency. Although this agrees with the findings of the present study, it is unlikely that muscle fatigue levels could have considerably influenced the results due to the time elapsed between sessions.

Females presented higher smiling consistency than males. Previous studies support this finding [30] and show that females are more comfortable to pose a smile [49] and they also smile more frequently than males [50]. In addition, females have a 10% larger proportional smile width than males [34], indicating that they tend to smile with more intensity. There are several social implications stemming from these findings. A large smile is a sign of a happier face, which is perceived as more trustworthy compared to less happy or angry face [51]. Galinsky et al. recruited a group of northern Europeans and recorded their perception of trustworthiness when observing a series of male and female stimulus characters with various facial expressions. Their result indicated that both, male and female participants perceived smiling female faces as more trustworthy than male ones [52]. It is also well known that females tend to smile more than males in a variety of social conditions [53] with several theories being proposed to explain this observation. Culturally, females are expected to appear pleasant and to smile more than men. At the same time, men are expected to smile less in order to appear more dominant and intimidating. Based on the ‘biosocial female choice theory’ (BFC), females show a mating preference towards men who appear that can provide multifaceted support during their reproductive years [54,55]. From a biological point of view, an increase in testosterone production creates a shift in brain function that counteracts smiling. This has been confirmed in animal studies showing a masculinization of sex-specific behaviour after exposure to testosterone [56,57] and in humans, where a negative correlation between circulating testosterone and smiling has been described [58]. It would thus not be an overstatement to support that historically, smiling is more associated to females than to males.

The use of geometric morphometrics to perform a shape analysis of the smile, as presented in this study, has not been used before. Johnston et al. evaluated the reproducibility of facial expressions in a sample of 30 young adults using two-dimensional facial photos. The face was described with landmarks that were directly placed on the faces of the participants prior to image acquisition. They reported that average differences between two consecutive smiles were approximately 1 mm. However they also reported that the method included considerable amount of error in landmark placement [28].

Ju et al. analysed three-dimensional images using motion curves to study the reproducibility of facial expressions and reported high reproducibility of the maximum smile [29]. However, they referred to the expression of the entire face. Out of the 23 landmarks that were used to describe the face, only 6 represented the structures of the lips. Thus, it is likely that the results were influenced more by the changes in the upper face than the perioral tissues. Given that the upper face is easier to control upon smiling [8,14], the results could have been confounded by the stability of the upper face expression upon smiling, mediating the outcomes on the reproducibility of the lip and smile morphology. Sawyer et al. also used three-dimensional images and described the face with 25 landmarks to test the reproducibility of a series of facial expressions [30]. While intrasession reproducibility was high, the authors report that after one month there were significant differences among subjects upon smiling. Nevertheless, these differences were 1.2 mm, which can be considered of small magnitude. As mentioned before, this indicates that the results are more affected by the change in upper face landmarks than the changes of the smile itself. Furthermore, it needs to be considered that the use of traditional facial landmarks, as described by Farkas [59] entails inherent error, since most soft tissue landmarks do not fulfil the requirements of actual anatomical landmarks, which by definition describe locations where there is a visible change in local anatomy, such the extremes of a structure or the crossing point of two types of tissue or texture [38]. The only facial points that fulfil this definition are the stomia and the vermillion borders of the lips. In the present study, the stomia were considered as fixed anatomical landmarks and the lip vermillia were described with curves along the anatomical border of the lips. Therefore, the error of landmark placement was reduced increasing the reliability of the results. The robustness of the methodology is further supported by the notably larger sample size compared to previous, related studies. Given that a sample of 50 is considered adequate in geometric morphometric studies that assess differences between independent groups, the current study population provides a solid sample particularly consisting of paired data [60,61].

As a secondary aim, the association between smile consistency and self-perceived smile attractiveness was evaluated, but was found to be insignificant. The initial presumption was that individuals who find their smile attractive would be more confident to smile, and thus they would smile more often, with the latter perhaps leading to a higher consistency in smiling expressions. However, this was not confirmed by the results. Assuming that social smiling is a cognitive function, it is learned during the course of a lifetime and is dependent on cultural and social parameters. Based on the environmental influences, an individual may have developed a habitual smiling performance, with different facial activity compared to a spontaneous smile [24,45]. Self-perception of attractiveness, on the other hand is a more perceptual process, less controlled by cognitive brain mechanisms. Decisions on facial attractiveness are usually reached within milliseconds and are more related to intrinsic functions related to social, sexual and mating preferences [62,63]. Within this biological context, the habitual smile of an individual might not likely be related to their opinion regarding their own smile attractiveness, which may explain the present results. The attractiveness of the smile is influenced by a variety of physical and psychosocial traits, including skin tone and texture [64,65], smile dimensions [34], smile masculinity/femininity and symmetry [63,66], features related to tooth exposure and the presence of black triangles [67], as well as self-esteem [68,69]. Most of these parameters are human-specific traits, which is in line with the notion that perception of attractiveness is a function not influenced by cognitive acts such as the repeatability of the smile.

The present study provides valuable information for restorative dentists, orthodontists, plastic and maxillofacial surgeons, and other specialists who focus on improving facial and smile aesthetics of their patients. Based on the findings, when patients are asked to perform a social smile in order to assess tooth position and exposure, as well as appearance of the lips, they are very consistent in performing this facial movement. This enables accurate diagnosis and, thus, targeted treatment planning regarding the smile and the associated dental and soft-tissue structures in cases that this is indicated. It also enables the proper interpretation of the outcome of an intervention or the assessment of changes over time when serial facial images of the same individual are compared. Furthermore, the results supplement the current literature in the field of psychosocial sciences that study facial expressions, their relationship to human interactions, and their effect on the perception of facial attractiveness.

## 5. Limitations

This study assessed smile repeatability focusing on lip morphology in a group of young adults. Although the sample size is significantly larger compared to other studies that addressed the same research question, the results are influenced by the inherent characteristics of the population. As muscle tone, skin texture and facial appearance change with time [70], it is not known whether the results would be different in an older population. It must also be considered that in some cases involuntary facial expressions might have influenced the results [71], nevertheless, previous research has not found clinically relevant deviations caused by small muscle contractions in adults [72].

The study also focuses on lip morphology during social smile, without considering other anatomical structures whose shape is also altered during smile, such as the perioral tissues surrounding the lips, the cheeks, or the eyes. Special surgical techniques are, for example, often used to alter eye morphology in order to improve overall facial attractiveness [73,74]. We followed this approach since the lips are the main anatomical structures that are highly adaptable and define smile shape and the amount of dental and gingival exposure. Therefore, the study focused on the lips as the main anatomical structure defining smile shape and related to smile consistency. Another large component of a smile are the teeth. In addition to orthodontics, other dental specialties play an important role in improving smile attractiveness by maintaining or restoring the natural dentition or by providing restorative options in cases where the natural dentition has been compromised. For this purpose, dental implants offer a stable and viable solution with good patient satisfaction even in cases with high aesthetic demands [75,76,77,78]. Although teeth are also directly related to the smile and contribute significantly to smile attractiveness, they were not assessed in the present manuscript since teeth remain mostly morphologically stable in the short-term, and thus, do not affect smile consistency. Furthermore, 3D images do not depict the teeth well. For the reasons stated above, the present study investigated the smile through the assessment of lip morphology only and although this might be considered a limitation, it focused the interest on a well-defined structure that is also the most relevant to the study outcome. A broader morphological assessment would mostly regard facial shape during smile rather than the smile shape itself. This is also interesting (although from another perspective) and it could comprise the topic of another study.

## 6. Conclusions

The shape of lips when performing a social smile is highly repeatable in young adults with mean deviations not exceeding 1.5 mm in most cases. Smile repeatability is more profound in females, who also perceive their smile as more attractive. Smile attractiveness, however, was not associated to the repeatability of the smile.

## Figures and Tables

**Table 1:** Differences between males and females in smile consistency and self-perceived smile attractiveness.

		Mean	Standard Deviation	*p*-Value
Average distance between smile landmarks	Females (*n* = 57)	0.95	0.27	0.024
	Males (*n* = 36)	1.09	0.32	
Self-perceived smile attractiveness	Females (*n* = 57)	72.84	17.88	0.026
	Males (*n* = 36)	64.72	15.25	

## Data Availability

The data is available on request from the corresponding author. This excludes images of participants, who have not provided consent to publish their facial images.

## References

[1] Ekman P., Rosenberg E.L. (2005). What the Face Reveals—Basic and Applied Studies of Spontaneous Expression Using the Facial Action Coding System (FACS).

[2] Shore D.M., Heerey E.A. (2011). The Value of Genuine and Polite Smiles. Emotion.

[3] Pallett P., Martinez A. (2014). Beyond the Basics: Facial Expressions of Compound Emotions. J. Vis..

[4] Stefani E.D., Ardizzi M., Nicolini Y., Belluardo M., Barbot A., Bertolini C., Garofalo G., Bianchi B., Coudé G., Murray L. (2019). Children with Facial Paralysis Due to Moebius Syndrome Exhibit Reduced Autonomic Modulation during Emotion Processing. J. Neurodev. Disord..

[5] Ishii L.E., Nellis J.C., Boahene K.D., Byrne P., Ishii M. (2018). The Importance and Psychology of Facial Expression. Otolaryng. Clin. N. Am..

[6] Trotman C.A., Faraway J., Hadlock T., Banks C., Jowett N., Jung H.J. (2018). Facial Soft-Tissue Mobility: Baseline Dynamics of Patients with Unilateral Facial Paralysis. Plast. Reconstr. Surg-Glob. Open.

[7] Girard J.M., Cohn J.F., Mahoor M.H., Mavadati S., Rosenwald D.P. Social Risk and Depression: Evidence from Manual and Automatic Facial Expression Analysis. Proceedings of the 2013 10th IEEE International Conference and Workshops on Automatic Face and Gesture Recognition (FG).

[8] Guo H., Zhang X.-H., Liang J., Yan W.-J. (2018). The Dynamic Features of Lip Corners in Genuine and Posed Smiles. Front. Psychol..

[9] Calvo M.G., Gutiérrez-García A., Avero P., Lundqvist D. (2013). Attentional Mechanisms in Judging Genuine and Fake Smiles: Eye-Movement Patterns. Emotion.

[10] Lien J.J.-J., Kanade T., Cohn J.F., Li C.-C. (2000). Detection, Tracking, and Classification of Action Units in Facial Expression. Robot Auton. Syst..

[11] Li Y., Shi Z., Zhang H., Luo L., Fan G. (2018). Commentary: The Dynamic Features of Lip Corners in Genuine and Posed Smiles. Front. Psychol..

[12] Yasuda K., Nakano H., Yamada T., Albougha S., Inoue K., Nakashima A., Kamata Y., Sugiyama G., Tajiri S., Sumida T. (2019). Identifying Differences Between a Straight Face and a Posed Smile Using the Homologous Modeling Technique and the Principal Component Analysis. J. Craniofac. Surg..

[13] Reed L.I., Sayette M.A., Cohn J.F. (2007). Impact of Depression on Response to Comedy: A Dynamic Facial Coding Analysis. J. Abnorm. Psychol..

[14] Park S., Lee K., Lim J.-A., Ko H., Kim T., Lee J.-I., Kim H., Han S.-J., Kim J.-S., Park S. (2020). Differences in Facial Expressions between Spontaneous and Posed Smiles: Automated Method by Action Units and Three-Dimensional Facial Landmarks. Sensors.

[15] Schmidt K.L., Swearingen J.M.V., Levenstein R.M. (2005). Speed, Amplitude, and Asymmetry of Lip Movement in Voluntary Puckering and Blowing Expressions: Implications for Facial Assessment. Mot. Control..

[16] Niedenthal P.M., Mermillod M., Maringer M., Hess U. (2010). The Simulation of Smiles (SIMS) Model: Embodied Simulation and the Meaning of Facial Expression. Behav. Brain Sci..

[17] Perusquía-Hernández M., Ayabe-Kanamura S., Suzuki K. (2019). Human Perception and Biosignal-Based Identification of Posed and Spontaneous Smiles. PLoS ONE.

[18] Hess U., Kleck R.E. (1990). Differentiating Emotion Elicited and Deliberate Emotional Facial Expressions. Eur. J. Soc. Psychol..

[19] Ekman P. (2003). Darwin, Deception, and Facial Expression. Ann. N. Y. Acad. Sci..

[20] Duchenne G.-B. (1990). The Mechanism of Human Facial Expression.

[21] Lübbers H.-T., Medinger L., Kruse A.L., Grätz K.W., Obwegeser J.A., Matthews F. (2012). The Influence of Involuntary Facial Movements on Craniofacial Anthropometry: A Survey Using a Three-Dimensional Photographic System. Br. J. Oral Maxillofac. Surg..

[22] Ekman P., O’Sullivan M. (2006). From Flawed Self-assessment to Blatant Whoppers: The Utility of Voluntary and Involuntary Behavior in Detecting Deception. Behav. Sci. Law.

[23] Sonneville L.M.J.D., Verschoor C.A., Njiokiktjien C., het Veld V.O., Toorenaar N., Vranken M. (2002). Facial Identity and Facial Emotions: Speed, Accuracy, and Processing Strategies in Children and Adults. J. Clin. Exp. Neuropsychol. Neuropsychol. Dev. Cogn. Sect..

[24] Krumhuber E., Kappas A. (2005). Moving Smiles: The Role of Dynamic Components for the Perception of the Genuineness of Smiles. J. Nonverbal. Behav..

[25] Briñol P., DeMarree K.G., Smith K.R. (2010). The Role of Embodied Change in Perceiving and Processing Facial Expressions of Others. Behav. Brain Sci..

[26] Helwig N.E., Sohre N.E., Ruprecht M.R., Guy S.J., Lyford-Pike S. (2017). Dynamic Properties of Successful Smiles. PLoS ONE.

[27] Gadassi R., Mor N. (2016). Confusing Acceptance and Mere Politeness: Depression and Sensitivity to Duchenne Smiles. J. Behav. Exp. Psy..

[28] Johnston D.J., Millett D.T., Ayoub A.F., Bock M. (2003). Are Facial Expressions Reproducible?. Cleft Palate-Craniofacial J..

[29] Ju X., O’leary E., Peng M., Al-Anezi T., Ayoub A., Khambay B. (2016). Evaluation of the Reproducibility of Nonverbal Facial Expressions Using a 3D Motion Capture System. Cleft Palate-Craniofacial J..

[30] Sawyer A.R., See M., Nduka C. (2009). Assessment of the Reproducibility of Facial Expressions with 3-D Stereophotogrammetry. Otolaryngol.-Head Neck Surg..

[31] O’Doherty J., Winston J., Critchley H., Perrett D., Burt D.M., Dolan R.J. (2003). Beauty in a Smile: The Role of Medial Orbitofrontal Cortex in Facial Attractiveness. Neuropsychologia.

[32] Furl N., Gallagher S., Averbeck B.B. (2012). A Selective Emotional Decision-Making Bias Elicited by Facial Expressions. PLoS ONE.

[33] Kanavakis G., Halazonetis D., Katsaros C., Gkantidis N. (2021). Facial Shape Affects Self-Perceived Facial Attractiveness. PLoS ONE.

[34] Horn S., Matuszewska N., Gkantidis N., Verna C., Kanavakis G. (2021). Smile Dimensions Affect Self-Perceived Smile Attractiveness. Sci. Rep..

[35] Liu J., Zhang C., Cai R., Yao Y., Zhao Z., Liao W. (2021). Accuracy of 3-dimensional stereophotogrammetry: Comparison of the 3dMD and Bellus3D facial scanning systems with one another and with direct anthropometry. Am. J. Orthod. Dentofac. Orthop..

[36] Dindaroğlu F., Kutlu P., Duran G.S., Görgülü S., Aslan E. (2016). Accuracy and reliability of 3D stereophotogrammetry: A comparison to direct anthropometry and 2D photogrammetry. Angle Orthod..

[37] Lübbers H.T., Medinger L., Kruse A., Grätz K.W., Matthews F. (2010). Precision and accuracy of the 3dMD photogrammetric system in craniomaxillofacial application. J. Craniofac. Surg..

[38] Gunz P., Mitteroecker P. (2013). Semilandmarks: A Method for Quantifying Curves and Surfaces. Hystrix-Ital. J. Mammal..

[39] Bookstein F.L. (1997). Landmark Methods for Forms without Landmarks: Morphometrics of Group Differences in Outline Shape. Med. Image Anal..

[40] Rohlf F.J., Slice D. (1990). Extensions of the Procrustes Method for the Optimal Superimposition of Landmarks. Syst. Zool..

[41] Aitken R.B. (1969). Measurement of Feelings Using Visual Analogue Scales. Proc. R. Soc. Med..

[42] Kanavakis G., Silvola A.-S., Halazonetis D., Lähdesmäki R., Pirttiniemi P. (2021). Profile Shape Variation and Sexual Dimorphism amongst Middle-Aged Northern Europeans. Eur. J. Orthodont..

[43] Ekman P., Friesen W., Hager J. (2002). FACS Investigator’s Guide (The Manual on CD Rom).

[44] Krumhuber E., Manstead A.S.R., Cosker D., Marshall D., Rosin P.L. (2009). Effects of Dynamic Attributes of Smiles in Human and Synthetic Faces: A Simulated Job Interview Setting. J. Nonverbal Behav..

[45] Schmidt K.L., Ambadar Z., Cohn J.F., Reed L.I. (2006). Movement Differences between Deliberate and Spontaneous Facial Expressions: Zygomaticus Major Action in Smiling. J. Nonverbal Behav..

[46] Gosselin P., Beaupré M., Boissonneault A. (2002). Perception of Genuine and Masking Smiles in Children and Adults: Sensitivity to Traces of Anger. J. Genet. Psychol..

[47] Gunnery S.D., Hall J.A., Ruben M.A. (2013). The Deliberate Duchenne Smile: Individual Differences in Expressive Control. J. Nonverbal Behav.

[48] Brach J.S., Van Swearingen J. (1995). Measuring fatigue related to facial muscle function. Arch. Phys. Med. Rehab..

[49] Walder J.F., Freeman K., Lipp M.J., Nicolay O.F., Cisneros G.J. (2013). Photographic and Videographic Assessment of The Smile: Objective and Subjective Evaluations of Posed and Spontaneous Smiles. Am. J. Orthod. Dentofac. Orthop..

[50] Hinsz V.B., Tomhave J.A. (1991). Smile and (Half) the World Smiles with You, Frown and You Frown Alone. Personal. Soc. Psychol. Bull..

[51] Oosterhof N.N., Todorov A. (2009). Shared Perceptual Basis of Emotional Expressions and Trustworthiness Impressions from Faces. Emotion.

[52] Galinsky D.F., Erol E., Atanasova K., Bohus M., Krause-Utz A., Lis S. (2020). Do I Trust You When You Smile? Effects of Sex and Emotional Expression on Facial Trustworthiness Appraisal. PLoS ONE.

[53] LaFrance M., Hecht M.A., Paluck E.L. (2003). The Contingent Smile: A Meta-Analysis of Sex Differences in Smiling. Psychol. Bull..

[54] Dodd D.K., Russell B.L., Jenkins C. (1999). Smiling in School Yearbook Photos: Gender Differences from Kindergarten to Adulthood. Psychol. Rec.

[55] Ellis L. (2006). Gender Differences in Smiling: An Evolutionary Neuroandrogenic Theory. Physiol. Behav..

[56] Compaan J.C., van Wattum G., Ruiter A.J.H., de Oortmerssen G.A. (1993). van Koolhaas, J.M.; Bohus, B. Genetic Differences in Female House Mice in Aggressive Response to Sex Steroid Hormone Treatment. Physiol. Behav..

[57] Thornton J., Goy R.W. (1986). Female-Typical Sexual Behavior of Rhesus and Defeminization by Androgens given Prenatally. Horm. Behav..

[58] Dabbs J.M. (1997). Testosterone, Smiling, and Facial Appearance. J. Nonverbal Behav..

[59] Farkas L.G., Deutsch C.K. (1996). Anthropometric Determination of Craniofacial Morphology. Am. J. Med. Genet..

[60] Cardini A., Elton S. (2007). Sample size and sampling error in geometric morphometric studies of size and shape. Zoomorphology.

[61] Cardini A., Seetah K., Barker G. (2015). How many specimens do I need? Sampling error in geometric morphometrics: Testing the sensitivity of means and variances in simple randomized selection experiments. Zoomorphology.

[62] Willis J., Todorov A. (2006). First Impressions: Making up Your Mind after a 100-Ms Exposure to a Face. Psychol. Sci..

[63] Little A.C., Jones B.C., DeBruine L.M. (2011). Facial Attractiveness: Evolutionary Based Research. Philos. Trans. R. Soc. B Biol. Sci..

[64] Samson N., Fink B., Matts P.J., Dawes N.C., Weitz S. (2010). Visible Changes of Female Facial Skin Surface Topography in Relation to Age and Attractiveness Perception. J. Cosmet. Dermatol..

[65] Fink B., Matts P.J., Brauckmann C., Gundlach S. (2018). The Effect of Skin Surface Topography and Skin Colouration Cues on Perception of Male Facial Age, Health and Attractiveness. Int. J. Cosmet. Sci..

[66] Grammer K., Fink B., Moller A.P., Thornhill R. (2003). Darwinian Aesthetics: Sexual Selection and the Biology of Beauty. Biol. Rev..

[67] der Geld P.V., Oosterveld P., Heck G.V., Kuijpers-Jagtman A.M. (2007). Smile Attractiveness. Self-Perception and Influence on Personality. Angle Orthod..

[68] Fleming J.S., Courtney B.E. (1984). The Dimensionality of Self-Esteem: II. Hierarchical Facet Model for Revised Measurement Scales. J. Personal. Soc. Psychol..

[69] Oikawa H., Oikawa H., Sugiura M., Sugiura M., Sekiguchi A., Sekiguchi A., Tsukiura T., Tsukiura T., Miyauchi C.M., Miyauchi C.M. (2012). Self-Face Evaluation and Self-Esteem in Young Females: An FMRI Study Using Contrast Effect. Neuroimage.

[70] Windhager S., Mitteroecker P., Rupić I., Lauc T., Polašek O., Schaefer K. (2019). Facial Aging Trajectories: A Common Shape Pattern in Male and Female Faces Is Disrupted after Menopause. Am. J. Phys. Anthropol..

[71] Brons S., Darroudi A., Nada R., Bronkhorst E.M., Vreeken R., Berge S.J., Maal T., Kuijpers-Jagtman A.M. (2019). Influence of Involuntary Facial Expressions on Reproducibility of 3D Stereophotogrammetry in Children with and without Complete Unilateral Cleft Lip and Palate from 3 to 18 Months of Age. Clin. Oral Investig..

[72] Hermann N.V., Darvann T.A., Larsen P., Lindholm P., Andersen M., Kreiborg S. (2016). A Pilot Study on the Influence of Facial Expression on Measurements in Three-Dimensional Digital Surfaces of the Face in Infants with Cleft Lip and Palate. Cleft Palate-Craniofacial J..

[73] Hollander M.H.J., Schortinghuis J., Vissink A., Jansma J., Schepers R.H. (2020). Aesthetic outcomes of upper eyelid blepharoplasty: A systematic review. Int. J. Oral Maxillofac. Surg..

[74] Botti G., Botti C., Rossati L., Gualdi A., Nocini P., Nocini R., Bertossi D. (2019). “Dynamic Canthopexy” Drill Hole Canthal Repositioning. Aesthet. Surg. J..

[75] Gherlone E.F., Capparé P., Pasciuta R., Grusovin M.G., Mancini N., Burioni R. (2016). Evaluation of resistance against bacterial microleakage of a new conical implant-abutment connection versus conventional connections: An in vitro study. New Microbiol..

[76] Paolone G. (2017). Direct composites in anteriors: A matter of substrate. Int. J. Esthet. Dent..

[77] Ramani R.S., Bennani V., Aarts J.M., Choi J.J.E., Brunton P.A. (2020). Patient satisfaction with esthetics, phonetics, and function following implant-supported fixed restorative treatment in the esthetic zone: A systematic review. J. Esthet Restor Dent..

[78] Wittneben J.G., Wismeijer D., Brägger U., Joda T., Abou-Ayash S. (2018). Patient-reported outcome measures focusing on aesthetics of implant- and tooth-supported fixed dental prostheses: A systematic review and meta-analysis. Clin. Oral Implants Res..

